# Retrospective, controlled observational case study of patients with central retinal vein occlusion and initially low visual acuity treated with an intravitreal dexamethasone implant

**DOI:** 10.1186/s12886-016-0363-5

**Published:** 2016-10-27

**Authors:** Sibylle Winterhalter, Gerrit Alexander vom Brocke, Daniel Pilger, Annabelle Eckert, Juliane Schlomberg, Anne Rübsam, Matthias Karl Klamann, Enken Gundlach, Tina Dietrich-Ntoukas, Antonia Maria Joussen

**Affiliations:** Department of Ophthalmology, Camus Virchow- Klinikum, Charité-University Medicine Berlin, Augustenburger Platz 1, 13353 Berlin, Germany

**Keywords:** Central retinal vein occlusion, Corticosteroids, Intravitreal dexamethasone implant, Macular edema, Visual acuity

## Abstract

**Background:**

Patients with initially low visual acuity were excluded from the therapy approval studies for retinal vein occlusion. But up to 28 % of patients presenting with central retinal vein occlusion have a baseline BCVA of less than 34 ETDRS letters (0.1). The purpose of our study was to assess visual acuity and central retinal thickness in patients suffering from central retinal vein occlusion and low visual acuity (<0.1) in comparison to patients with visual acuity (≥0.1) treated with Dexamethasone implant 0.7 mg for macular edema.

**Methods:**

Retrospective, controlled observational case study of 30 eyes with macular edema secondary to central retinal vein occlusion, which were treated with a dexamethasone implantation. Visual acuity, central retinal thickness and intraocular pressure were measured monthly. Analyses were performed separately for eyes with visual acuity <0.1 and ≥0.1.

**Results:**

Two months post intervention, visual acuity improved only marginally from 0.05 to 0.07 (1 month; *p* = 0,065) and to 0.08 (2 months; *p* = 0,2) in patients with low visual acuity as compared to patients with visual acuity ≥0.1 with an improvement from 0.33 to 0.47 (1 month; *p* = 0,005) and to 0.49 (2 months; *p* = 0,003). The central retinal thickness, however, was reduced in both groups, falling from 694 to 344 μm (1 month; *p* = 0.003,) to 361 μm (2 months; *p* = 0,002) and to 415 μm (3 months; *p* = 0,004) in the low visual acuity group and from 634 to 315 μm (1 month; *p* < 0,001) and to 343 μm (2 months; *p* = 0,001) in the visual acuity group ≥0.1. Absence of visual acuity improvement was related to macular ischemia.

**Conclusions:**

In patients with central retinal vein occlusion and initially low visual acuity, a dexamethasone implantation can lead to an important reduction of central retinal thickness but may be of limited use to increase visual acuity.

**Electronic supplementary material:**

The online version of this article (doi:10.1186/s12886-016-0363-5) contains supplementary material, which is available to authorized users.

## Background

Retinal vein occlusions (RVO) are the most common primary vascular diseases of the retina [1, 2]. Branch retinal vein occlusions (BRVO) are more common than central retinal vein occlusions (CRVO) with a prevalence of 0.6–1.1 versus 0.1–0.4 % in the general population [3, 4]. The spontaneous disease course is often poor especially in CRVO and hemi CRVO but also in BRVO. The majority of patients with retinal vein occlusion suffer from visual deterioration because of macular edema (ME). Quinlan [5] found in 1990 that only 13 to 17 % of patients with CRVO had a significant visual acuity (VA) gain without treatment. The visual prognosis is thereby dependent on the initial VA and the degree of retinal ischemia [6]. An initial VA of <20/200 (<0.1) seems to be connected with a low VA prognosis. During the natural course of CRVO patients will suffer a medium VA impairment of 1 line VA chart [6].

The sham injection groups of the newer RVO therapy trials [7, 8] provide also data of the spontaneous disease course. Patients of the CRVO sham group lost in average 1 to 2 letters after 6 months of follow up during the GENEVA trial. In contrast to this the BRVO sham group gained 5 letters in average after 6 months [7, 8]. The GENEVA trial [7, 8] excluded RVO patients with a BCVA of less than 34 ETDRS letters (0.1). But up to 28 % of patients presenting with CRVO have a baseline BCVA of less than 34 ETDRS letters and more than 80 % have a poor final VA if untreated [6].

The CRVO study group found in 1995 that grid pattern photocoagulation may reduce the leakage but without VA gain in CRVO patients with ME [9]. Since the Dexamethasone implant Ozurdex® (DEX implant) and the VEGF inhibitors Ranibizumab and Aflibercept were approved by the European Medicines Agency (EMA) in 2010, 2011 and 2013 for the treatment of ME secondary to RVO, the therapeutic options improved. However it is not clear if these new drugs have the capacity to improve the visual prognosis of patients with initial low VA (<0.1). In this study CRVO patients with low VA have been treated with DEX implantation. The aim was to evaluate the outcome after intervention in this specific patient population.

## Methods

In this retrospective, controlled, observational case study 30 eyes of 29 patients with ME secondary to CRVO were treated with a 700 μg sustained delivery, bioerodable Dexamethasone intravitreal implant (DEX implant; Ozurdex®; Allergan, Inc., Irvine, CA) between November 2010 and July 2011. All patients gave their informed consent and all procedures were in concordance with the tenets of the Declaration of Helsinki. All patients were treated on label so that no ethics approval was needed for the retrospective chart review.

### Patients

Patients with ME >300 μm secondary to CRVO were included into the study. Patients with known glaucoma were only included in case of well controlled intraocular pressure (IOP). Some patients received topical glaucoma medication after the CRVO occured because neuroprotection is suspected for e.g. carbonic anhydrase inhibiting eye drops [10, 11]. Exclusion criteria were any ocular condition that was able to interfere with potential visual improvement and a pre-treatment with a DEX implantation at any time point. Analyses were performed separately for eyes with visual acuity <0.1 (15 eyes) and ≥0.1 (15 eyes).

### Follow- up and treatment

The baseline examinations included assessment of VA and IOP, complete ophthalmologic examination and central retinal thickness (CRT) measured by Spectral Domain Optical Coherence Tomography (SD-OCT) (Heidelberg Eye Explorer Version 6.011.0, Heidelberg Spectralis, Heidelberg Engineering, Heidelberg, Germany) before DEX implantation. Follow-up was conducted every 4 weeks over a time period of 6 months including VA, SD-OCT and complete ophthalmologic examination. A retreatment was performed mostly after 5 to 6 months if necessary so that this report refers only till month 5 after DEX implantation. Decimal visual acuity was converted into ETDRS letters with a VA conversion chart [12] to compare our results with the newer RVO therapy trials.

Side effects of the DEX implant were monitored by measuring the IOP every 4 weeks. Fluorescein angiography (FA) was performed in cases of missing contraindications (renal failure, reduced general condition, dye allergies) before DEX implantation or during the disease course to visualise macular and retinal perfusion. The FA procedure followed the multi-field protocol used by the Central Vein Occlusion Study Group, with standard 5- view angiography [13] using the HRA Spectralis (Heidelberg Eye Explorer Version 6.011.0, Heidelberg Engineering, Heidelberg, Germany) with a 55° lens and the patients were asked to look in all directions to visualize the periphery. In cases of significant peripheral retinal ischemia patients were treated with panretinal photocoagulation to avoid neovascularisation and rubeotic secondary glaucoma. The peripheral retina was regarded as ischemic if ischemia amounted more than 10 disc diameters. Retreatment with DEX implantation or Ranibizumab was considered in cases of recurrent ME.

### Statistics

Data were checked for normal distribution and results are expressed in mean ± standard deviation (SD). Normally-distributed variables were compared with a matched paired sample *t*-test. Numeric variables that were not normally distributed were compared with the matched paired Wilcoxon signed rank test. The Chi^2^- test was used to compare the gender differences between the groups. The results were regarded as statistically significant if p was below 0.05. Statistics were performed with SPSS (Version 21.0.0.0, IBM, United States).

## Results

### Patients

#### Low visual acuity group

Six men and nine women with a baseline visual acuity <0.1 (15 eyes) were treated with a DEX implantation. The mean time after CRVO was 9 (±6.9) months in this group and the mean age of patients 72.5 (±9) years. Four patients (26.7 %) were pretreated with bevacizumab. Six patients (40 %) received topical glaucoma medication to protect the optic nerve head. One patient was diagnosed with chronic open angle glaucoma.

#### Control group

The control group consisted of seven men and eight women with a baseline visual acuity ≥0.1 (15 eyes) and a mean time after CRVO of 9 (±7.3) months. The mean age of the control group amounted 72.3 (±9.9) years. Two patients (13.3 %) were pretreated with bevacizumab and one patient (6.7 %) was pretreated with bevacizumab and triamcinolone. Eight patients (53.3 %) received topical glaucoma medication at baseline. Two of these patients were diagnosed with chronic open angle glaucoma before treatment.

All DEX implantations were performed under standardized conditions (operating theatre, topical anaesthesia, sterile conditions, DEX implantation in tunnel technique in 3.5 mm distance of the limbus) without implantation related complications.

### Visual acuity

#### Low visual acuity group

**Fig. 1:**
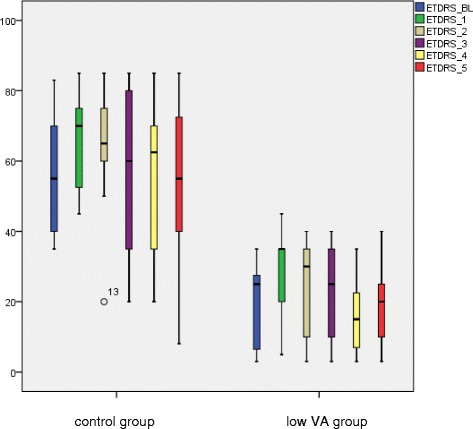
Visual acuity course of the low visual acuity group in comparison to the control group after DEX implantation expressed in ETDRS letters with standard deviation

**Fig. 2:**
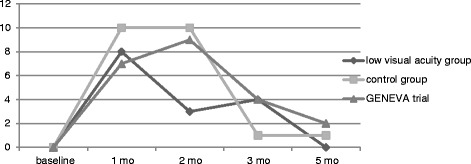
Mean BCVA improvement in numbers of letters of the low visual acuity and control group in comparison to the GENEVA trial

Throughout the follow up the distance VA of the low VA group improved compared to baseline (Table 1). VA improvement showed a weak effect but without statistical significance at any time point. The conversion into ETDRS letters is seen in Table 1, Figs. 1 and 2. The Additional file 1a-e shows in more detail the ETDRS letter change compared to baseline during month 1 to 5 after DEX implantation. A ≥15 letter improvement was achieved in 40 % of patients and a ≥10 letter improvement in 53.3 % of patients at any time point.

**Table 1:** VA and CRT in 15 CRVO patients with baseline VA <0.1

	Visual acuity	ETDRS letters (VA)	CRT (μm)
Baseline (±SD)	0.05 (±0.03)	20 (±12)	694 (±301)
1 mo follow-up	0.07 (±0.05)	28 (±13)	344 (±127)
*P*-value	0.065	0.1	0.003*
2 mo follow-up	0.08 (±0.04)	23 (±14)	361 (±226)
*P*-value	0.2	0.23	0.002*
3 mo fyollow-up	0.07 (±0.04)	24 (±13)	415 (±224)
*P*-value	0.14	0.19	0.004*
4 mo follow-up	0.05 (±0.03)	17 (±12)	534 (±299)
*P*-value	0.31	0.8	0.094
5 mo follow-up	0.06 (±0.04)	20 (±12)	665 (±340)
*P*-value	0.93	0.82	0.14

(*p-values with statistical significance)

#### Control group

In the control group, the distance VA assessment showed an improvement at month 1 (*p* = 0.005) and month 2 (*p* = 0.003) (Table 2). The conversion into ETDRS letters is shown in Table 2, Figs. 1 and 2 with the same effect from month 1 (*p* = 0.002) to month 2 (*p* = 0.013). The Additional file 2a-e shows in more detail the ETDRS letter change compared to baseline during month 1 to 5 after DEX implantation. A ≥15 letter improvement was achieved in 46.7 % of patients and a ≥10 letter improvement in 73.3 % of patients at any time point.

**Table 2:** VA ad CRT in 15 CRVO patients with baseline VA ≥ 0.1

	Visual acuity	ETDRS letters (VA)	CRT (μm)
Baseline (±SD)	0.33 (±0.27)	55 (±17)	634.38 (±130.40)
1 mo follow-up	0.47 (±0.27)	65 (±13)	314.92 (±77.88)
*P*-value	0.005*	0.002*	<0.001*
2 mo follow-up	0.49 (±0.29)	65 (±25)	343.27 (±73.89)
*P*-value	0.003*	0.013*	0.001*
3 mo follow-up	0.44 (±0.37)	56 (±25)	483.17 (±255.16)
*P*-value	0.24	1	0.18
4 mo follow-up	0.4 (±0.35)	55 (±24)	534.13 (±191.86)
*P*-value	0.49	0.97	0.24
5 mo follow-up	0.39 (±0.39)	54 (±26)	490.5 (±76.15)
*P*-value	0.13	0.77	0.23

(*p-values with statistical significance)

### Central retinal thickness

#### Low visual acuity group

The CRT assessed by SD-OCT was reduced compared to baseline with strong evidence for an effect during the first (*p* = 0.003), second (*p* = 0.002) and third month (*P* = 0.004) after DEX implantation (Table 1).

#### Control group

In the control group the CRT was reduced compared to baseline again with a strong evidence for an effect from month 1 (*p* < 0.001) to month 2 (*p* = 0.001) (Table 2).

### Fluorescein angiography and panretinal photocoagulation

FA was performed in 14 eyes of the low VA group and 11 eyes of the control group before (18 eyes) or after DEX implantation (seven eyes). The remaining five patients did not receive a FA because of contraindications.

#### Low visual acuity group

**Fig. 3:**
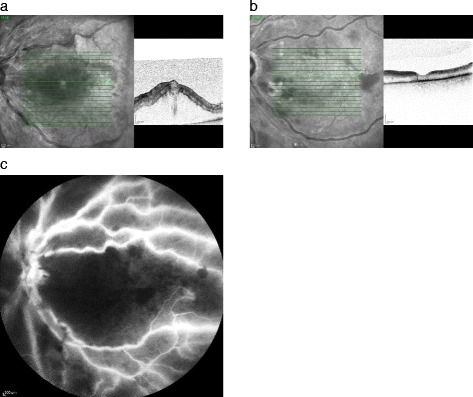
**a** – **c** 81 year old lady, who suffered of CRVO with low VA on her left eye and was treated with a DEX implantation 2 months after the occlusion. **a** VA counted 3 ETDRS letters at baseline with a CRT of 924 μm measured with the SD- OCT. **b** VA augmented to 35 ETDRS letters (0.1) 1 month after treatment with a reduced CRT of 214 μm. **c** VA gain was limited because the macula and retina were totally ischemic as seen in FA

FA showed macular ischemia in 64.3 % of patients (Table 3, Fig. 3) shows a case report). Peripheral retinal ischemia was present in 84.6 % of patients during FA likewise (Table 3, Fig. 3). One of the patients with macular ischemia did not have peripheral retinal ischemia and three other patients with perfused macular showed peripheral retinal ischemia. In total 57.1 % of patients showed macular and peripheral retinal ischemia at the same time point. The periphery of one patient could not be evaluated because of poor quality. All patients received panretinal photocoagulation (100 %).

**Table 3:** Macular and peripheral retinal ischemia of the low VA group in comparison to the control group

Ischemia visualized by FA	Low VA group	Control group
Macular	64.3 %	18.2 %
Peripheral retinal	84.6 %	36.4 %

#### Control group

Macular ischemia was present in 18.2 % of patients (Table 3, 63.6 % perfused, 18.2 % not evaluable). Peripheral retinal ischemia could be detected in 36.4 % of patients (Table 3, 36.4 % perfused, 27.3 % not evaluable). Only one patient showed macular and peripheral retinal ischemia at the same time point. Eight patients were treated with panretinal photocoagulation (53.3 %).

### Intraocular pressure (IOP)

#### Low visual acuity group

The median IOP was: 14.3 ± 2.4 mmHg (baseline); 18.5 ± 2.6 mmHg (month 1); 19.3 ± 4 mmHg (month 2); 17.1 ± 1.6 mmHg (month 3); 16.7 ± 3 mmHg (month 4) and 17.8 ± 9.9 mmHg (month 5). The IOP lowering medication had to be intensified in one patient with open angle glaucoma and in three further patients (26.7 %).

#### Control group

In this group the median IOP was: 14.1 ± 5.2 mmHg (baseline); 18.9 ± 8.2 mmHg (month 1); 19.1 ± 7.4 mmHg (month 2); 16.8 ± 3.5 mmHg (month 3); 16.4 ± 4 mmHg (month 4) and 13 ± 2.6 mmHg (month 5). One patient with open angle glaucoma and 5 further patients needed an intensified IOP lowering medication (40 %).

The IOP raised to ≥25 mmHg in 20 % of patients and a ≥10 mmHg increase of IOP from baseline at 60 days was seen in 13.3 % of patients. Three patients suffered of IOP decompensation with 40 mmHg in month 1 (control group) and 38 mmHg in month 2 (control group) after DEX implantation. One patient with an intraocular pressure of 35 mmHg in month 5 showed rubeotic secondary glaucoma (low VA group) and was treated with retinal cryocoagulation during follow-up.

### Retreatment

A retreatment was performed in 6 patients (40 %) of the low visual acuity group 4 to 9 months after DEX implantation with a medium retreatment time of 7 months.

Patients of the control group were retreated in 53.3 % (8 patients) 5 to 8 months after DEX implantation with a medium retreatment time of 5.9 months.

## Discussion

Our retrospective controlled study was performed to evaluate VA gain after DEX implantation in patients with initially low VA <0.1 because CRVO is often related to limited visual prognosis [5, 6, 14] and the GENEVA trial [7, 8] excluded RVO patients with a BCVA of less than 34 ETDRS letters (0.1). Our results show that patients with initially low VA can profit of DEX implantation despite weak evidence for an effect: 40 % of patients achieved a ≥15 letter improvement and 53.3 % of patients a ≥10 letter improvement at any time point.

In contrast patients of the control group experienced VA amelioration with strong evidence for an effect from month 1 (*p* = 0.005) to month 2 (*p* = 0.003). This is in concordance to other studies [15, 16]. The ETDRS letter gain of the control group was higher than in the low VA group with 46.7 % of patients who gained ≥15 letters and 73.3 % of patients who improved with ≥10 letters at any time point. During the GENEVA trial [7, 8] a ≥15 letter improvement in BCVA from baseline was seen in up to 30 % in the 0.7 mg DEX group and at least a ≥10 letter improvement in up to 55 % of eyes.

In opposition to our results Dinah [17] described in 19 CRVO patients with a baseline BCVA of less than 34 ETDRS letters that 70 % of patients gained ≥15 letters. In their study, up to a third of patients achieved a BCVA of 55 ETDRS letters after DEX implantation. In our study no patient of the low VA group achieved 55 ETDRS letters and only 1 patient achieved 45 ETDRS letters and another one 40 ETDRS letters.

The limited VA gain in our low VA group was related to macular ischemia which amounted up to 64.3 %.

In clinical experience macular ischemia is often not visible during FA in cases of diffuse edema. After ME resolution, the ischemia becomes apparent in FA. In contrast to the low VA group macular ischemia was present in 18.2 % in the control group. A high correlation is known between low baseline VA and presence or development of ischemia [6]. Therefore, the benefit of patients with low VA and macular ischemia seems to be limited after DEX implantation. As Dinah et al. [17] did not evaluate FA, the retinal perfusion status of the cohort is not known.

Another recent study of Parodi examined DEX implants for macular edema secondary to ischemic retinal vein occlusions [18]. Fifteen patients with CRVO and macular and retinal ischemia were included in this study with a follow up of 12 months. The median ETDRS letter score of these patients was 10 at baseline. This is in contrast to our case series of 15 patients with low VA with a median ETDRS letter score of 20 at baseline. The CRVO patients of Parodis group gained 10 ETDRS letters after 1 month which is comparable with a letter gain of 8 ETDRS letters after 1 month in our patients. But in contrast to our patients, who worsened again at month 2, Parodis CRVO patients showed a steady letter gain till 37 ETDRS letters (0.1) at 12 months, which might be relevant for the patient but is limited too, through a mean number of 2.8 DEX implantations. All 15 patients of Parodis study were diagnosed with macular and retinal ischemia, which was the inclusion criterion. The inclusion criterion of our patients was a VA <0.1 to evaluate VA expectations in low VA patients because no informations are available of the approval studies for these patients. Parodis CRVO patients showed a CRT reduction from 749 μm at baseline to 363 μm at the 12 month examination. This is comparable to our patients with a CRT reduction from 694 μm to the minimum CRT of 344 μm on month 1 after DEX implantation. A reevaluation of the perfusion status of Parodis patients would have been interesting because of the steady letter gain till month 12. There are hints that aggressive blockade of VEGF with ranibizumab prevents the worsening of retinal non perfusion, promotes reperfusion and eliminates a positive feedback loop in patients with RVO [19].

Our results are in concordance with the study of Maggio [20] in which 21 eyes with CRVO, 20 eyes with BRVO and two eyes with hemiretinal vein occlusion (HRVO) were treated with a DEX implantation. They found that only the nonischemic CRVO subgroup had a statistically significant improvement in BCVA and CRT. The ischemic CRVO group did not improve significantly in BCVA in spite of significant improvements in CRT [20]. The CRT of our low VA group was reduced as well with strong evidence for an effect from month 1 to 3 (*p* = 0.003; *p* = 0.002; *p* = 0.004) despite a weak evidence for an effect concerning BCVA improvement. Other studies confirm that macular ischemia is a significant negative factor for VA improvement as described for intravitreal Bevacizumab for ME due to BRVO [21].

In our study the highest mean BCVA improvement in numbers of letters was similar to the GENEVA trial but our patients reached their highest letter gain earlier and dropped down earlier than the patients of the GENEVA trial. Effects of Dexamethasone implants are known in the literature as early as 7 days after injection regarding VA improvement and CRT reduction [22, 23]. Probably, an earlier treatment and retreatment might have been associated with a more favourable outcome.

Concerning the safety results a ≥10 mmHg increase of IOP from baseline was seen in 13.3 % of patients compared to 12.6 % of patients at day 60 of the GENEVA trial [7, 8]. In total the topical glaucoma- medication had to be intensified in 33.3 % of patients of our study whereas 25.5 % of patients began an IOP lowering medication during the masked phase of the GENEVA trial [7, 8].

A combination therapy of vascular endothelial growth factor (VEGF) inhibitors and DEX implants might have been associated with better VA results because the medium CRT was higher than 300 μm after DEX implantation in both groups of our study at any time point. Regarding the literature a combination therapy of vascular endothelial growth factor (VEGF) inhibitors and DEX implants improved significantly the VA outcome in BRVO patients but not in CRVO patients [24]. However, the approval studies for VEGF inhibitors [25–27] to treat ME due to RVO showed a higher ETDRS letter gain than the GENEVA trial [7, 8]. A combination therapy may lead to higher VA gain despite fewer necessary injections than with VEGF inhibitor therapy alone [28–31] and may be therefore cost-effective [32]. Despite this a combination therapy would not resolve the macular and retinal ischemia which was associated to limited final VA in our study. Otherwise CRVO patients of the CRYSTAL study with macular ischemia showed a VA improvement of 11.6 ETDRS letters under 3 primary Ranibizumab injections and following PRN treatment after 12 months which was comparable to patients without macular ischemia (12.1 ETDRS letters) [33]. But the inclusion criterion of the CRYSTAL study was a baseline BCVA of 19 to 73 ETDRS letters (0.05–0.5) whereas the inclusion criterion for our low VA group was a VA <34 ETDRS letters (<0.1). So a more severe macular ischemia is suspected in our patients than that of the patients of the CRYSTAL study or Ranibizumab might be more effective in CRVO patients with macular ischemia than DEX implants.

Our study has a number of limitations. First, this was a retrospective study, and therefore we do not have full control over confounding factors. Also, our results have to be interpreted with caution due to the small sample size which resulted in low statistical power. Hence, interpretation of our findings should consider observed trends and not entirely rely on statistical significance. These trends are largely similar to the trends bowered in larger studies such as the GENEVA trial (Fig. 2) which makes a chance finding unlikely. A further limitation results in the mean duration of CRVO which was 9 months in the low VA group and could have resulted in a lower VA gain. But it has to be said that the mean duration of CRVO was 9 months in the control group as well which showed a similar ETDRS letter gain than the patients of the GENEVA trial. This study was performed shortly after DEX implants were approved in Europe for the treatment of RVO, so the duration of CRVO was longer in some patients and some patients were pretreated with bevacizumab. But the baseline characteristics of the control group were similar to the low VA group so that our results seem to be realistic. Our results showed as well, that some patients should have been retreated earlier as described before [16], which was a learning effect.

## Conclusion

VA prognosis is limited after DEX implantation in CRVO patients with initial low VA because of macular ischemia. Despite the limited VA prognosis we would recommend to treat patients due to the fact that these patients showed a slight benefit in our study. However, patients should be informed about the limited VA prognosis to avoid unrealizable expectations. The results may help to prevent risks and burden for the patients and costs for the health care system in patients with CRVO and low VA.

## Additional files

Additional file 1: a-c) waterfall plots of the ETDRS letter change 1 to 5 months after DEX implantation compared to baseline of the low VA group. (DOCX 48 kb)
Additional file 2: a-c) waterfall plots of the ETDRS letter change 1 to 5 months after DEX implantation compared to baseline of the control group. (DOCX 48 kb)

## Abbreviations

BCVA: Best corrected visual acuity
BRVO: Branch retinal vein occlusion
CRT: Central retinal thickness
CRVO: Central retinal vein occlusion
DEX implant: Dexamethasone implant
EMA: European medicines agency
ETDRS: Early treatment diabetic retinopathy study
FA: Fluorescein angiography
IOP: Intraocular pressure
ME: Macular edema
RVO: Retinal vein occlusion
SD: Standard deviation
SD-OCT: Spectral domain optical coherence tomography
VA: Visual acuity
VEGF: Vascular endothelial growth factor
