# Supplementary figures and images for: Retrospective, controlled observational case study of patients with central retinal vein occlusion and initially low visual acuity treated with an intravitreal dexamethasone implant
Source: BMC Ophthalmol. 2016 Oct 27;16:187. doi: 10.1186/s12886-016-0363-5 (PMC5081977; doi:10.1186/s12886-016-0363-5)

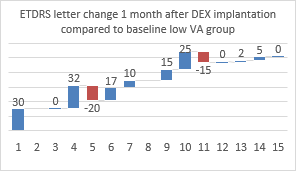
 1a


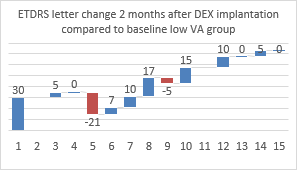
 1b


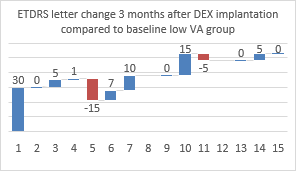
 1c


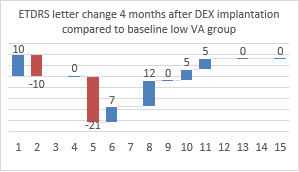
 1d


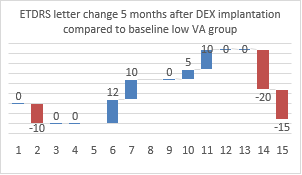
 1e

Supplement: Additional file 1: — a-c) waterfall plots of the ETDRS letter change 1 to 5 months after DEX implantation compared to baseline of the low VA group. (DOCX 48 kb) [file 12886_2016_363_MOESM1_ESM.docx]

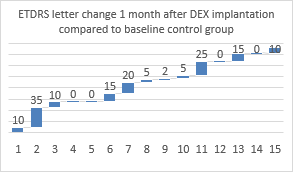
 2a


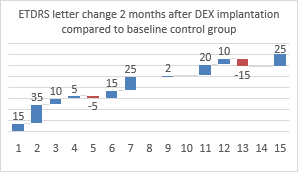
 2b


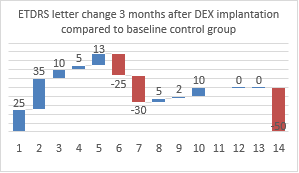
 2c


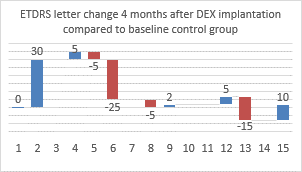
 2d


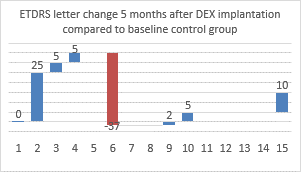
 2e

Supplement: Additional file 2: — a-c) waterfall plots of the ETDRS letter change 1 to 5 months after DEX implantation compared to baseline of the control group. (DOCX 48 kb) [file 12886_2016_363_MOESM2_ESM.docx]
